# The robustness of glenohumeral centering measurements in dependence of shoulder rotation and their predictive value in shoulders with rotator cuff tears

**DOI:** 10.1007/s00256-022-04159-6

**Published:** 2022-08-25

**Authors:** Rainer J. Egli, Emma C. J. Widmer, Manuel Waltenspül, Samy Bouaicha, Reto Sutter

**Affiliations:** 1 Radiology, Balgrist University Hospital, University of Zurich, Zurich, Switzerland; 2 Department for Diagnostic, Interventional, and Paediatric Radiology, Bern University Hospital, University of Bern, Freiburgstrasse 15, 3010 Bern, Switzerland; 3 Department of Orthopedics, Balgrist University Hospital, University of Zurich, Zurich, Switzerland

**Keywords:** Glenohumeral centering, Acromiohumeral distance, Rotator cuff tear, Shoulder

## Abstract

**Objective:**

De-centering of the shoulder joint on radiographs is used as indicator for severity of rotator cuff tears and as predictor for clinical outcome after surgery. The objective of the study was to assess the effect of malrotation on glenohumeral centering on radiographs and to identify the most reliable parameter for its quantification.

Subjects and methods.

In this retrospective study (2014–2018), 249 shoulders were included: 92 with imaging-confirmed supra- and infraspinatus tears (*rupture*; 65.2 ± 9.9 years) and 157 without tears (*control*; 41.1 ± 13.0 years). On radiographs in neutral position and external rotation, we assessed three radiographic parameters to quantify glenohumeral centering: acromiohumeral distance (ACHD), craniocaudal distance of the humeral head and glenoid center (Deutsch), and scapulohumeral arch congruity (Moloney). Non-parametric statistics was performed.

**Results:**

In both positions, only the distance parameters ACHD (< 0.5 mm) and Deutsch (< 1 mm) were comparable in the two study groups *rupture* and *control*. Comparing the parameters between the study groups revealed only ACHD to be significantly different with a reduction of more than 2 mm in the *rupture* group. Among the parameters, ACHD ≤ 6 mm was the only cut-off discriminating *rupture* (12–21% of the shoulders with ACHD ≤ 6 mm) and *control* (none of the shoulders with ACHD ≤ 6 mm). Ninety percent of shoulders with ACHD ≤ 6 mm presented with a massive rotator cuff tear (defined as ≥ 67% of the greater tuberosity exposed).

**Conclusion:**

Glenohumeral centering assessed by ACHD and Deutsch is not affected by rotation in shoulders with and without rotator cuff tear. An ACHD ≤ 6 mm has a positive predictive value of 90% for a massive rotator cuff tear.

## Introduction

Rotator cuff tears of the shoulder often result from an acute intense trauma or a combination of tendon degeneration and repetitive minor trauma [1, 2]. The prevalence is approximately 10% in individuals younger than 20 years and > 60% in individuals older than 80 years [3]. Rotator cuff tears can be debilitating due to restriction of movement and pain. They are often accompanied by cranial migration of the humeral head attributed to the imbalance of the cranializing force of the deltoid muscle and the caudalizing effect of the rotator cuff and the long biceps tendon [4]. De-centering of the glenohumeral joint measured on conventional radiographs has been used as an indicator for the severity of rotator cuff tears and as a prognostic factor for the success of intended surgical approaches [5–7]. Therefore, accurate measuring of glenohumeral centering is key when being used for clinical decision-making.

Conventional radiographs of the shoulder are the first line of diagnostic imaging in patients with shoulder pain. Several radiographic parameters are available to address glenohumeral centering: acromiohumeral distance, distance of the center of the humeral head from the center of the glenoid in cranio-caudal direction according to Deutsch [8], and congruity of the scapulohumeral arch [9]. These parameter measurements are based on a two-dimensional projection of a three-dimensional anatomic relation. As such, the measurements are prone to radiographic projection errors or patient-related conditions. The latter is of special importance, since patients may not be able to take the correct positions for diagnostic radiographs due to muscle contractions and pain. It has been recently shown in a cadaver study that malrotation of the shoulder resulted in significant and clinically relevant differences in measurement parameters of the scapula [10]. There is no data available in the literature, however, on the influence of malrotation on the assessment of glenohumeral centering on conventional radiographs. We hypothesized that glenohumeral centering is dependent on the rotation of the humeral head and that this difference is more pronounced in patients with posterosuperior rotator cuff tears since shoulder centering is dependent on an intact rotator cuff. The two objectives of our study were (1) to analyze the influence of malrotation on radiographic projection and (2) to identify the most suitable parameter to measure glenohumeral centering on conventional radiographs in a clinical setting.

## Subjects and methods

### Study design and patient selection

The study was approved by the institutional review board (2020–01298). All patients included in this study signed a general consent on the usage of health-related data for research purposes.

**Fig. 1:**
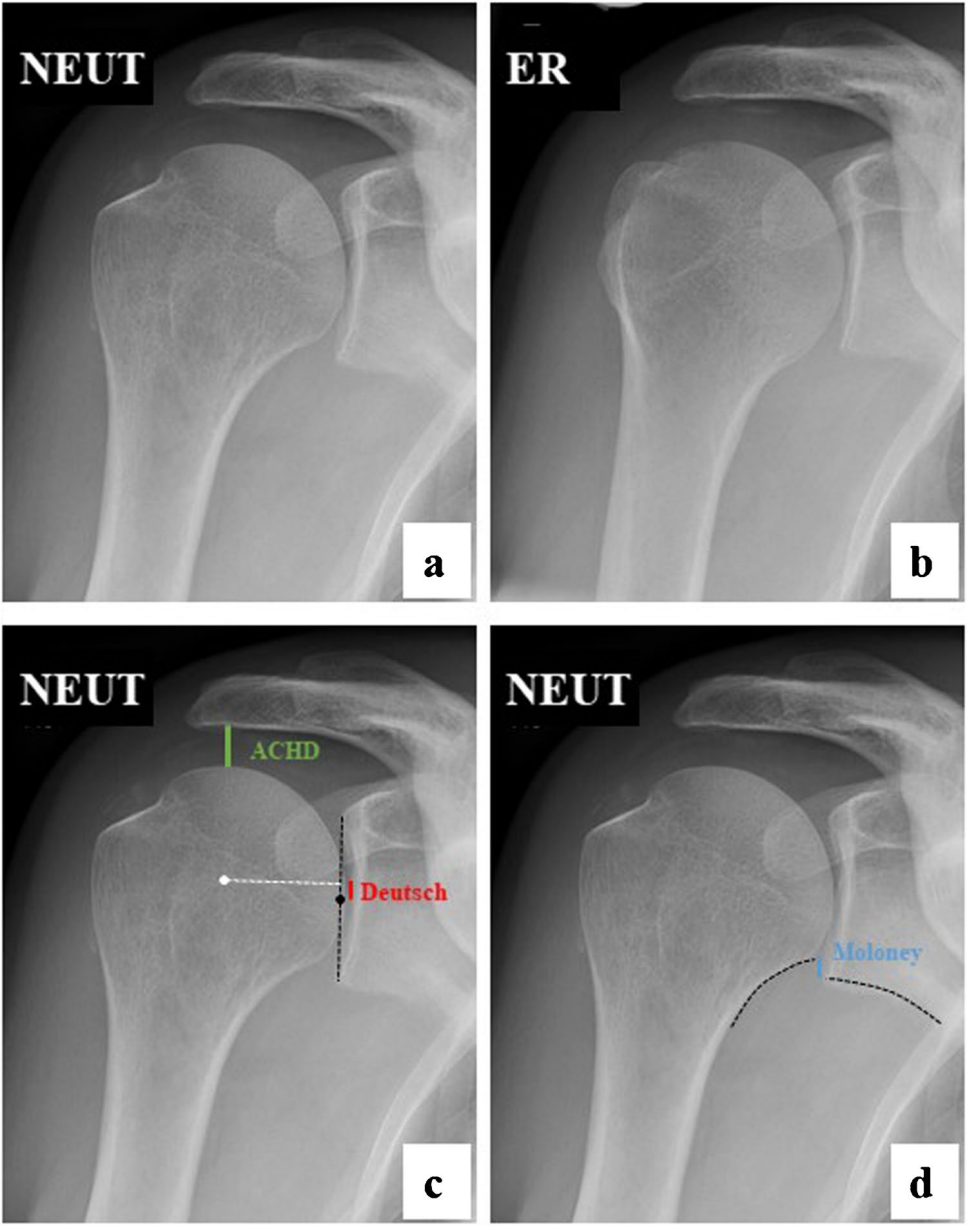
Oblique anteroposterior radiographs of a right shoulder in neutral position (NEUT) and in external rotation (ER). **a**, **b** The rotation of the humerus alters the projected shapes of the humeral head and the calcar, influencing the measurements for glenohumeral centering. **c** Glenohumeral centering was quantified by the acromiohumeral distance (ACHD), by the distance of the center of the humeral head to the center of the glenoid in cranio-caudal direction according to Deutsch, and **d** by the congruity of the scapulohumeral arch, known as the Moloney line

In this retrospective study, the radiology information system (RIS) of our department was reviewed for patients older than 18 years who received two oblique anteroposterior shoulder radiographs (Grashey view) with both the humerus in neutral position (termed as NEUT) and 45° external rotation (termed as ER) (Fig. 1 a–b) over a period of four and a half years (January 2014 to May 2018) and also received an MRI (magnetic resonance imaging)- or CT (computed tomography)-arthrography within 90 days of the radiographs. A total of 3089 shoulders were identified.

#### Study groups

The study comprised two groups: (i) patients with a rotator cuff tear (*rupture* group), and (ii) patients with an intact rotator cuff (*control* group). In the *rupture* group, 92 shoulders were included (65.2 ± 9.9 years), and 157 in the *control* group (41.1 ± 13.0 years).

The reports of the MRI- or CT-arthrography were screened for exclusion and inclusion criteria as detailed below.

#### Patient exclusion criteria—*rupture* and *control* group

Patients with a prior surgical history of the shoulder (open and arthroscopically), fractures (including Hill-Sachs and osseous Bankart lesions), prior shoulder dislocation, clinical or radiological signs of frozen shoulder, and patients with severe cuff arthropathy (Hamada stages 4 and 5) [11] were excluded from both study groups.

#### Patient inclusion criteria—*rupture* group

Reports indicating a rotator cuff tear involving the supra- and infraspinatus tendon, also known as posterosuperior rotator cuff tear, and an intact or a subscapularis tendon tear involving not more than the upper third of the tendon resulted in 121 potential candidates. The MRI- and CT-arthrographies were analyzed for the following parameters: (i) extent of the rotator cuff tear in anteroposterior direction [12] (see below); (ii) tendon retraction according to Patte [13]; (iii) fatty infiltration of the muscle according to Goutallier [14]; and (iv) atrophy of the supraspinatus muscle by the presence of the tangent sign [15]. The extent of the rupture in anteroposterior direction was determined based on the method introduced in a recently published paper, which identified a denominator for the term “massive rotator cuff tear” using the Delphi-method [12]. The quantification is based on the percentage of the greater tuberosity exposed by the teared rotator cuff tendons, measured in the sagittal plane with a value of ≥ 67% defining the cut off for a “massive rotator cuff tear,” while a value below 67% was considered a “non-massive rotator cuff tear.” Based on these criteria, a total of 92 shoulders were included in the *rupture* group, and of those 65 shoulders (65/92, 71%) presented with a massive rotator cuff tear.

#### Patient inclusion criteria—*control* group

MRI or CT were analyzed to confirm an intact rotator cuff. A total of 157 shoulders were included in the *control* group.

### Analysis of the conventional radiographs

Measurements on oblique anteroposterior radiographs of 92 shoulders enrolled in the *rupture* group and 157 shoulders enrolled in the *control* group were independently performed by two readers: reader 1 (EW) is a final-year medical student with no previous experience in radiology; reader 2 (RE) is a board certified radiologist with 5-year experience and specializing in musculoskeletal radiology.

Glenohumeral centering was quantified by measuring three parameters (Fig. 1 c–d): (i) acromiohumeral distance (ACHD); (ii) distance of the center of the humeral head to the center of the glenoid in cranio-caudal direction according to Deutsch et al. [8] (in the following termed as Deutsch); and (iii) congruity of the scapulohumeral arch, known as the Moloney line [9] (in the following termed as Moloney). For Deutsch and Moloney, a positive value was given when the humeral head was cranially displaced in relation to the glenoid, a negative value when caudally displaced. Cut-off values based on the measurements of glenohumeral centering were established to discriminate between shoulders with and without a rotator cuff tear. Two different cut-off values were defined for each measurement: (i) the first value must not be reached by any control in both readers; (ii) the second value must not be reached by more than 5% of controls in both readers.

Additionally, the quality of the radiographic projection taken in NEUT position was rated by reader 2. Four radiographic criteria were analyzed for defining an appropriate radiograph, according to the qualitative analysis criteria termed “omometry” [10]: (i) anterior and posterior rim of the glenoid projects as one line; (ii) the coracoid process is in harmony with the superior glenoid rim; (iii) the diameter of the coracoid process is equal or larger than the acromial diameter; and (iv) the diameter of the acromion does not increase towards the acromio-clavicular joint. Each of the four parameters met counted as one point, resulting in a scale from 0 (poor projection) to 4 (optimal projection).

### Statistics

SPSS statistics was used for data analysis (IBM SPSS Statistics, Version 26 for Windows). Based on the results of the Kolmogorov–Smirnov test, all statistical analysis was performed with non-parametric tests.

Intraclass correlation (ICC) was calculated to assess interrater reliability. Two-way mixed measures checked for consistency. ICC values of < 0.50 were rated as poor, 0.50–0.75 as moderate, 0.75–0.90 as good, and > 0.90 as excellent [16].

Measures for glenohumeral centering (ACHD, Deutsch, and Moloney) were statistically assessed using the Wilcoxon signed rank test for the comparison of NEUT versus ER, and the Mann–Whitney *U* test for comparison of *control* versus *rupture*. Distribution of gender and side in *rupture* and *control* was assessed with non-parametric chi-square tests. Omometry and glenohumeral centering were compared using the Kruskal–Wallis test for non-parametric and independent variables. The correlation of massive rotator cuff tears with different MR-based parameters was done using Fisher’s exact test.

## Results

### Patient demographics

A total of 249 shoulders were enrolled in the study, 92 in the *rupture*, and 157 in the *control* group. The patients in *rupture* were significantly older compared to *control* (65.2 ± 9.9 and 41.1 ± 13.0 years, respectively, *p* < 0.001). There was a significant overrepresentation of males in *control*, but not in *rupture* (61.1% (*p* < 0.01) and 54.3%, respectively). In both groups, right shoulders were significantly overrepresented in *rupture* (72.8%, *p* < 0.001) and in *control* (58.6%, *p* < 0.05).

### Interrater correlation

The interrater correlation of the two readers for ACHD (pooled data from *rupture* and *control*) was rated excellent for both projections (NEUT 0.945, ER 0.916). For Deutsch, interrater correlation was rated moderate in NEUT and good in ER (NEUT 0.709, ER 0.782), and for Moloney good in NEUT and moderate in ER (NEUT 0.841, ER 0.737).

### Glenohumeral centering in dependence of rotation of the humeral head

Glenohumeral centering assessed by ACHD, Moloney, and Deutsch was significantly different in most comparisons between NEUT and ER projections (Table 1). However, the mean differences for ACHD were < 0.5 mm and for Deutsch < 1 mm for both readers and both groups. The mean differences for Moloney were more pronounced with apparent inferior translation of the humeral head in external rotation by 2.5–3.6 mm.

**Table 1:** Glenohumeral centering in dependence of rotation and the presence of a rotator cuff tear. *NEUT* neutral position, *ER* external rotation. Numbers are mean distances in mm and standard deviations. * *p* < 0.05, ** *p* < 0.005, *** *p* < 0.001, statistical comparison between NEUT and ER with non-parametric tests of dependent variables (Wilcoxon signed-rank test). ^‡^ *p* < 0.05, ^‡‡^ *p* < 0.005, ^‡‡‡^ *p* < 0.001, statistical comparison between NEUT of the *rupture* and *control* group with non-parametric tests of independent variables (Mann–Whitney *U* test)

		ACHD	Reader 1 Deutsch	Moloney	ACHD	Reader 2 Deutsch	Moloney
Control	NEUT	11.0 ± 2.3	− 0.4 ± 1.9	3.0 ± 3.0	11.2 ± 2.3	− 0.2 ± 1.8	1.2 ± 3.1
	ER	10.5 ± 2.1 ***	− 1.4 ± 1.9 ***	− 0.6 ± 3.3 ***	10.8 ± 2.0 **	− 0.5 ± 2.4	− 1.3 ± 3.3 ***
Difference (mean)		0.5	1.0	3.6	0.4	0.3	2.5
Rupture	NEUT	8.5 ± 2.5 ^‡‡^	− 0.5 ± 2.5	2.6 ± 4.3	9.1 ± 2.5 ^‡‡‡^	0.8 ± 2.2 ^‡‡‡^	2.6 ± 4.3 ^‡^
	ER	8.1 ± 2.2 *	− 0.3 ± 2.5	− 0.9 ± 4.3 ***	8.9 ± 2.3	0.6 ± 2.4	− 0.3 ± 4.0 ***
Difference (mean)		0.4	− 0.2	3.5	0.2	0.2	2.9

### Omometry and glenohumeral centering

Omometry scores were given to 21.6% (34/157, score 0), 18.5% (29/157, score 1), 24.2% (38/157, score 2), 18.5% (38/157, score 3), and 17.2% (27/157, score 4) of the NEUT projections in *controls*. A subgroup analysis of glenohumeral centering in dependence of the omometry score did only find a statistically significant difference for Deutsch in reader 1 (*p* = 0.037). Post hoc analysis after Bonferroni correction for multiple comparisons, however, did not find a statistically significant difference in any comparison, the lowest value calculated between omometry score 1 and 2 (*p* = 0.085). All other subgroup analyses did not show statistically significant differences for ACHD (*p* = 0.893 and 0.883 for reader 1 and reader 2, respectively), Deutsch (*p* = 0.113 for reader 2), and Moloney (*p* = 0.946 and 0.901 for reader 1 and reader 2, respectively).

### Glenohumeral centering in dependence of rotator cuff tear

Most of the measurements for glenohumeral centering indicated a cranial migration of the humeral head in the patients of the *rupture* group as compared to *control* in both positions NEUT and ER (Table 1). The mean value of the ACHD in NEUT position of the *rupture* group was significantly reduced compared to *control* in both readers by 2.5 and 2.1 mm, respectively. The mean differences in *rupture* compared to *control* in NEUT position for Deutsch were − 0.1 mm for reader 1 and 1 mm for reader 2, and for Moloney − 0.4 mm and 1.4 mm, with significant differences only found by reader 2 (Table 1).

Cut-off values based on the measurements of glenohumeral centering were established to discriminate between shoulders with and without a rotator cuff tear (Table 2). Two different cut-off values were defined for each measurement: (i) the first value must not be reached by any control in both readers; (ii) the second value must not be reached by more than 5% of controls in both readers. The cut-off values identified for ACHD were ≤ 6 mm and ≤ 7 mm, for Moloney ≥ 14 mm and ≥ 8 mm, and for Deutsch ≥ 8 mm and ≥ 4 mm, respectively.

**Table 2:** Cut-off values for glenohumeral centering as discriminator between patients with rotator cuff tears (rupture) and control patients (control). Numbers are absolute number of shoulders, with percentages in parenthesis. Fisher exact test, *p < 0.05, ** *p* < 0.01, *** *p* < 0.001

		Reader 1 rupture *n* = 92	Control *n* = 157	Reader 2 rupture *n* = 92	Control *n* = 157
ACHD	≤ 6 mm	19 (20.7%)***	0 (0%)	11 (12%)***	0 (0%)
	≤ 7 mm	31 (33.7%)***	2 (1.3%)	22 (23.9%)***	2 (1.3%)
Moloney	≥ 14 mm	0 (0%)	0 (0%)	0 (0%)	0 (0%)
	≥ 8 mm	9 (9.8%)*	5 (3.2%)	11 (12.0%)***	3 (1.9%)
Deutsch	≥ 8 mm	0 (0%)	0 (0%)	1 (1.1%)	0 (0%)
	≥ 4 mm	4 (4.3%)	6 (3.8%)	8 (8.7%)*	4 (2.5%)

The most striking differences were found for ACHD where a consistent significant over-representation of shoulders with rotator cuff tears and a reduced ACHD were measured (Table 2). In particular, 20.7% (19/92) of shoulders with a rotator cuff tear in reader 1 and 12% (11/92) in reader 2 presented with an ACHD ≤ 6 mm, while in none of the controls, an ACHD ≤ 6 mm was measured. The discrimination between shoulders with and without a rotator cuff tear was less evident for the chosen cut-off values for Moloney and Deutsch, with significant differences only for the less stringent values (< 5%) applied.

**Fig. 2:**
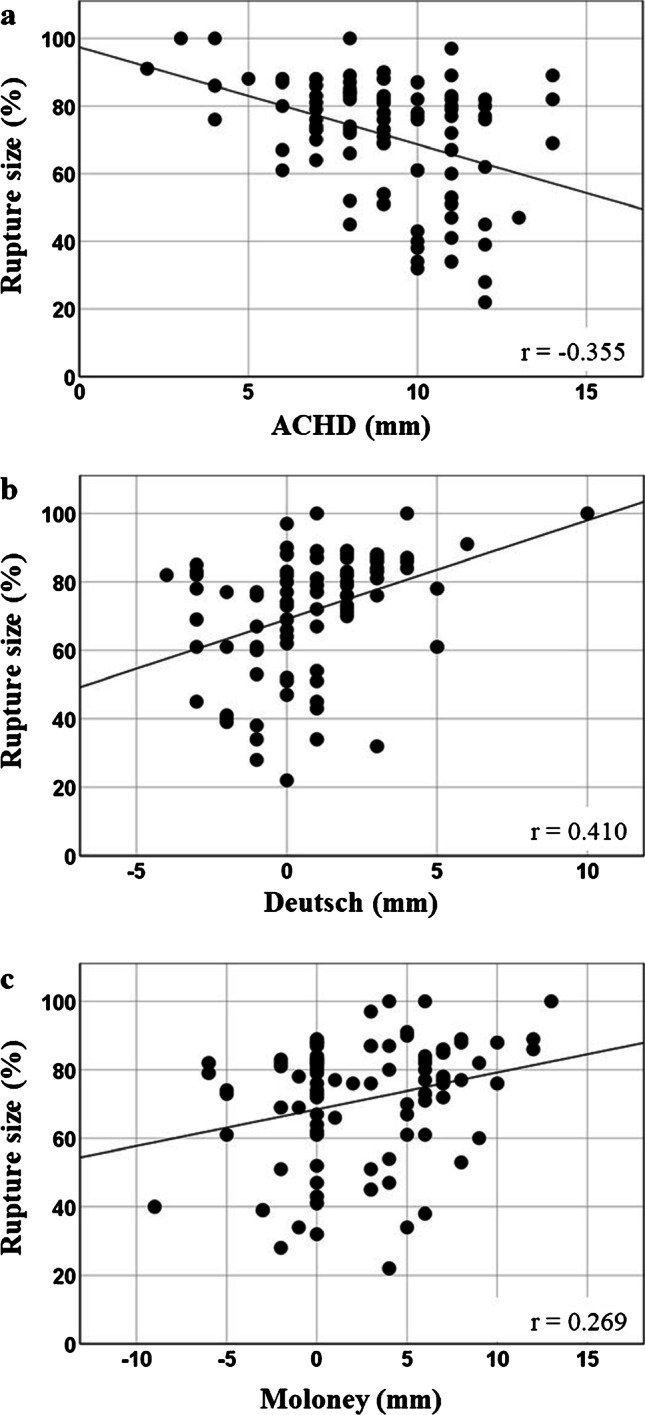
Correlation of the parameters for glenohumeral centering and the size of rotator cuff tear in patients of the *rupture* group (data from reader 2). **a** ACHD, **b** Deutsch, and **c** Moloney. Note that due to the measurement definitions, cranial migration of the humeral head manifests with a reduction of ACHD, but with an increased distance for Deutsch and Moloney

**Fig. 3:**
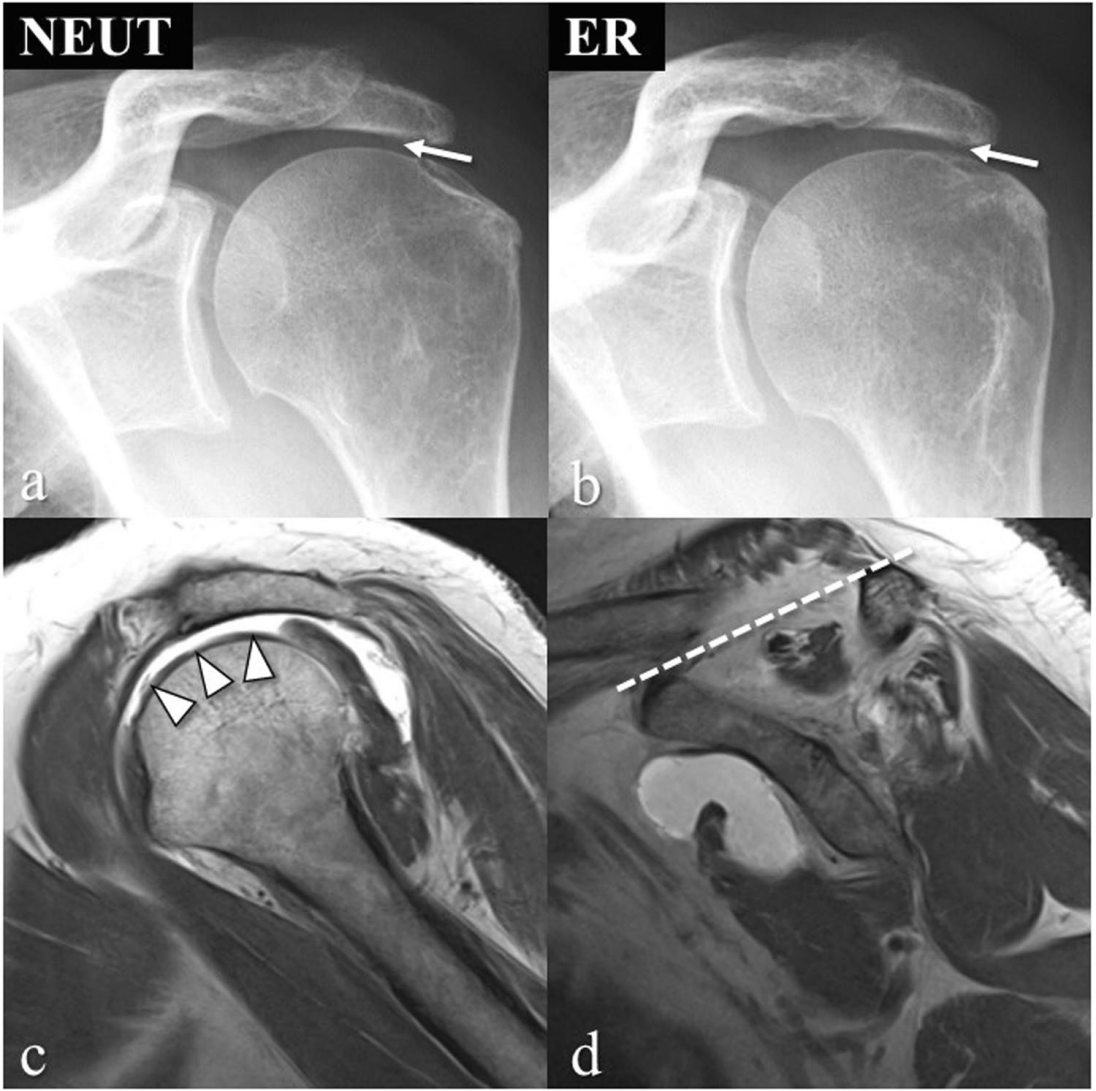
Massive rotator cuff tear in the left shoulder of a 56-year old woman. **a**, **b** Oblique anteroposterior radiograph in neutral position (NEUT) shows small acromiohumeral distance (ACHD) as indicated by the arrow, with no change in external rotation (ER). **c** Sagittal T1-weighted MR image depicts a complete tear of the supraspinatus tendon (arrowheads). **d** Sagittal T1-weighted MR image shows a positive tangent sign of an atrophied supraspinatus muscle; i.e., the muscle does not cross the dotted line connecting the superior border of the coracoid process to the superior border of the scapular spine

We adopted a quantitative measure for the size of the rotator cuff tear by assessing the extent of the greater tuberosity that is exposed due to tendon ruptures, with a value of ≥ 67% considered a massive tear [12]. A statistically significant, albeit, weak correlation was found between all three distance parameters and rupture size (Fig. 2). Out of the 92 shoulders from the *rupture* group, 65 fulfilled the characteristics of a massive tear. Shoulders with a massive rotator cuff tear showed a significantly reduced ACHD and increased Deutsch in both readers as compared to shoulders with a tear not considered to be massive, whereas no significant difference was found for Moloney (Fig. 3 and Table 3).

**Table 3:** Glenohumeral centering in patients with massive vs. non-massive rotator cuff tears. * *p* < 0.05, ** *p* < 0.01, *** *p* < 0.005. Spearman correlation to test correlation between glenohumeral centering and tear size. Statistical comparison between non-massive and massive tears with non-parametric tests of dependent variables (Mann–Whitney *U* test)

Cuff tear	*n* =	ACHD	Reader 1 Moloney	Deutsch	ACHD	Reader 2 Moloney	Deutsch
Non-massive	27	9.5 ± 2.0	1.41 ± 4.3	− 1.85 ± 1.6	10.2 ± 1.7	1.44 ± 3.1	− 0.3 ± 1.8
Massive	65	8.0 5.6 **	3.14 ± 4.3	− 0.0 ± 2.6***	8.7 ± 2.6**	3.14 ± 3.3	1.17 ± 2.4***
Correlation to tear size		− 0.321**	0.408***	0.284**	− 0.355**	0.410***	0.269*

We further analyzed whether a specific abnormality of the rotator cuff was associated with a reduced ACHD in shoulders with massive tears (Table 4). Of those 65 shoulders, 17 shoulders of reader 1 (26.2%) and 10 shoulders of reader 2 (15.4%) presented with an ACHD ≤ 6 mm. In the entire study group *rupture* (see again Table 2), 17 of 19 shoulders with an ACHD ≤ 6 mm (89.5%) in reader 1, and 10 of 11 shoulders (90.9%) in reader 2 had a massive rotator cuff tear. Due to the low number of shoulders with massive rotator cuff tears and a reduced ACHD, retraction according to Patte was dichotomized in two groups (either 0–2 or 3), and fatty infiltration according to Goutallier was also dichotomized in two groups (either 0–2 or 3–4). In both readers, a significant association of the presence of a tangent sign with an ACHD ≤ 6 mm was found. A significant correlation of fatty infiltration of the supraspinatus muscle with a reduced ACHD was established only by reader 1. There was no statistically significant association between patients with a reduced ACHD (≤ 6 mm) for tendon retraction and atrophy of the infraspinatus muscle.

**Table 4:** The association of rotator cuff abnormalities on MRI with a reduced ACHD (≤ 6 mm) in shoulders with massive rotator cuff tears: specific MRI abnormalities Fisher’s exact test. Significantly different parameters are highlighted in bold. *SSP* supraspinatus, *ISP* infraspinatus

		Reader 1			Reader 2	
	≤ 6 mm *n* = 17	≥ 7 mm *n* = 48	*p*	≤ 6 mm *n* = 10	≥ 7 mm *n* = 55	*p*
Retraction (Patte = 3)	82.0%	64.5%	0.229	80.0%	67.3%	0.711
Tangent sign	**76.4%**	**39.6%**	**0.012**	**90.0%**	**43.6%**	**0.044**
SSP atrophy (Goutallier ≥ 3)	**64.7%**	**29.2%**	**0.019**	60.0%	34.5%	0.165
ISP atrophy (Goutallier ≥ 3)	76.5%	47.9%	0.051	60.0%	54.5%	1.000

## Discussion

Tears of the rotator cuff can result in cranial migration of the humeral head due to muscular imbalance of the cranializing effect of the deltoid muscle and the caudalizing effect of the rotator cuff [4]. The extent of cranial decentering has been used as an indicator for the severity of rotator cuff tears and as a prognostic factor for the success of intended surgical treatment approaches [5–7]. Therefore, accurate measuring of glenohumeral centering is key when being used for clinical decision making. In the present study, we compared three different parameters to quantify glenohumeral centering on conventional oblique anteroposterior radiographs (Grashey view) taken in two different positions (neutral and external rotation of the humerus) in patients with and without rotator cuff tears. We showed that glenohumeral centering measured either by ACHD or Deutsch is robust and is independent on shoulder rotation. Comparing shoulders with and without rotator cuff tear revealed that only ACHD can discriminate between those two groups, and that an ACHD ≤ 6 mm reliably predicts a rotator cuff tear.

The interrater correlation was excellent when glenohumeral centering was assessed by ACHD, independent on the rotation of the humerus. The two other parameters, Deutsch und Moloney, presented with lower interrater correlations; however, they were still considered moderate to good. The lower interrater agreement can be explained by the measurement method. While measuring ACHD is based on generally well-identifiable landmarks, the method of Deutsch requires proper identification of the superior and inferior glenoid tubercle, which are often less clearly identifiable on radiographs in cases where the glenoid is not well projected. For Moloney, a continuity of the scapular-humeral arch line needs to be projected, which always leaves some room for interpretation. We further evaluated the effect of the quality of radiographic projection on the parameters of glenohumeral centering using omometry [10]. Since there were no statistically significant differences observed for ACHD, Deutsch, and Moloney in dependence on the quality of the projection, it is reasonable to infer that those parameters are not substantially biased by inferior radiographic projections.

Differences in radiographic projections are a known factor that influence the measured parameters on shoulder radiographs, as was recently demonstrated in 86 shoulders, where the ACHD was significantly lower in supine radiographs than in upright weight-bearing radiographs [17], confirming the results of a study with 166 shoulders comparing supine CT and standing CT [18]. Also, the radiographic beam angle influences the measured parameters of shoulder radiographs, as was shown in a comparison between CT and radiographs in 28 shoulders [19]. Furthermore, the acromiohumeral distance is different between radiographic projections and cross-sectional imaging, as has been described in a study of 34 shoulders with massive rotator cuff tears [20].

While the effect of different radiographic projections has been investigated by several studies, the effect of shoulder rotation on the measurements of glenohumeral centering so far has not been explored in the literature. We investigated the effect of shoulder rotation on the measurement of glenohumeral centering for two reasons: the first being that patients with rotator cuff tears may not be able to hold the arm in the required position, and the second to address whether the action of the rotator cuff can affect glenohumeral centering in patients with and without rotator cuff tears. Even though statistically significant differences were found in our study when comparing the NEUT versus the ER position for ACHD and Deutsch, those cannot be considered clinically relevant, as the differences were smaller than 1 mm. Those observations were not dependent whether shoulders were in the *rupture* or the *control* group. As a side note, the small differences in ACHD can be attributed to a slightly smaller projection of the humeral head by approximately 0.5 mm in ER as compared to NEUT (data not shown) as the humerus is not perfectly spherical [21]. The finding of the rather constant results for ACHD and Deutsch is in line with a previous sonography-based study where no differences for ACHD in internal rotation, neutral position, and external rotation were found in non-abducted arms [22]. In contrast to ACHD and Deutsch, measuring glenohumeral centering using Moloney suggests the humeral head being translated caudally during external rotation by 2.5–3.5 mm. However, this is mainly due to different projections of the calcar in the radiographs. In NEUT, the calcar is projected en face, but it is out of plane and overlaid by the humeral head in ER, leading to the wrong assumption of glenohumeral decentering.

Shoulders with rotator cuff tears presented with a significantly smaller ACHD mean value of about 2–2.5 mm as compared to shoulders without a tear. For Moloney and Deutsch only by reader 2, a significant cranial migration of the humeral head by ≤ 1.4 mm was measured in patients with a rotator cuff tear as compared to controls. This inconsistency among the readers is most probably due to the difficulties to identify the landmarks for Deutsch and Moloney as stated above. The finding of a reduced ACHD in shoulders with rotator cuff tear was well expected and has been shown in many studies [23–26]. As a further refinement, our data strongly suggest that an ACHD ≤ 6 mm is a realistic threshold to infer a rotator cuff pathology since all of the 152 control shoulders had an ACHD of ≥ 7 mm. On the opposite, an ACHD of ≥ 7 mm has no predictive value with respect to the state of the rotator cuff since > 80% of shoulders with a rotator cuff tear also fall within this range. For the methods of Deutsch and Moloney, no such stringent thresholds could be established separating shoulders from the *rupture* and the *control* group as there is substantial overlap of the values in both groups.

We adopted a measure to quantify the extent of a rotator cuff tear by assessing the percentage of the greater tubercle exposed by the cuff tear [12]. Significant, albeit weak, correlations were found between the extent of the tear and the parameters for glenohumeral centering, with larger tear sizes being associated with a larger cranial migration of the humeral head. It is important to recognize that for an individual patient, the extent of the rotator cuff tear does not allow predicting the extent of cranial migration of the humeral head, while an ACHD ≤ 6 mm does predict a massive rotator cuff tear (> 66% of the greater tubercle exposed) with a positive predictive value of 90%.

We correlated a reduced ACHD in the subpopulation of massive rotator cuff tears with other MR-based parameters of the rotator cuff. The only consistent significant correlation was found for the tangent sign, which is more frequently present in shoulders with a reduced ACHD (75–90% as compared to approximately 40%). Other parameters like tendon retraction or fatty infiltration of the muscles did not, or only inconsistently, reveal significant differences. A positive tangent sign is the result of a combination of a rotator cuff tear, tendon retraction, and muscle atrophy and may explain why the correlation of the tangent sign with a reduced ACHD stands out. This of course confirms the well-known observation that a positive tangent sign is linked to massive rotator cuff tears [15].

As a limitation of the study, the *control* and the *rupture* group were neither age- nor gender-matched. This does not invalidate the findings when shoulder rotation was tested against glenohumeral centering as those were compared within the same shoulder. A lack of difference in those comparisons between the *rupture* and *control* group further corroborates this statement. An age-related change in glenohumeral centering independent of rotator cuff tears may have affected the comparison between *control* and *rupture*. We did not find, however, any statistical significant correlation between age and glenohumeral centering in the *control group*.

To conclude, only ACHD and Deutsch are independent on rotation of the glenohumeral joint. ACHD is the only parameter measured allowing a statement on the integrity of the rotator cuff: an ACHD ≤ 6 mm reliably predicts a massive rotator cuff tear with a positive predictive value of 90%.
